# High-Quality Draft Genome Sequence of the Actinobacterium *Nocardia terpenica* IFM 0406, Producer of the Immunosuppressant Brasilicardins, Using Illumina and PacBio Technologies

**DOI:** 10.1128/genomeA.01391-16

**Published:** 2016-12-15

**Authors:** Anina Buchmann, Michael Eitel, Pierre Koch, Paul N. Schwarz, Evi Stegmann, Wolfgang Wohlleben, Marcin Wolański, Michał Krawiec, Jolanta Zakrzewska-Czerwińska, Carmen Méndez, Alma Botas, Luz Elena Núñez, Francisco Morís, Jesus Cortés, Harald Gross

**Affiliations:** a Department of Pharmaceutical Biology, Institute of Pharmaceutical Sciences, University of Tübingen, Tübingen, Germany; b German Centre for Infection Research (DZIF), Partner Site Tübingen, Tübingen, Germany; c Department of Medicinal and Pharmaceutical Chemistry, Institute of Pharmaceutical Sciences, University of Tübingen, Tübingen, Germany; d Department of Microbiology and Biotechnology, Interfaculty Institute of Microbiology and Infection Medicine, University of Tübingen, Tübingen, Germany; e Faculty of Biotechnology, University of Wrocław, Wrocław, Poland; f Ludwik Hirszfeld Insitute of Immunology and Experimental Therapy, Polish Academy of Sciences, Wrocław, Poland; g Departamento de Biología Funcional e Instituto Universitario de Oncología del Principado de Asturias, Universidad de Oviedo, Oviedo, Spain; h EntreChem S.L., Oviedo, Spain

## Abstract

The bacterium *Nocardia terpenica* IFM 0406 is known as the producer of the immunosuppressant brasilicardin A. Here, we report the completely sequenced genome of strain IFM 0406, which facilitates the heterologous expression of the brasilicardin biosynthetic gene cluster but also unveils the intriguing biosynthetic capacity of the strain to produce secondary metabolites.

## GENOME ANNOUNCEMENT

Actinobacteria of the genus *Nocardia* are often found to be human and animal pathogens that cause pulmonary, cutaneous and subcutaneous diseases (1–3). However, species in this genus also produce a plethora of potent bioactive natural compounds with therapeutic potential. For example, *Nocardia terpenica* IFM 0406 (formerly referred to as *Nocardia brasiliensis* IFM 0406) is known to produce the antifungal and immunosuppressant brasilinolides A–C (4, 5) and brasilicardins A−D (6–9). The immunosuppressant brasilicardin A is of particular interest because its potency is superior to today’s standard immunosuppressive drugs for organ transplantation, but it is less toxic, since it mediates its activity by a different mode of action (10). The development of this promising lead structure is currently hampered due to supply issues. The producer strain only shows a low production titer and is furthermore categorized as a biosafety-level 2 organism. Both of these facts make the fermentative production of brasilicardin A expensive and elaborate. Due to its complex stereochemistry, a total synthesis has not been achieved to date (11–13). Therefore, we want to overcome this hurdle by heterologous expression of the corresponding biosynthetic gene cluster (14) in a nonpathogenic producer strain. In order to obtain the full biosynthetic pathway, including the related precursor metabolic pathways, we sequenced the genome of *N. terpenica* IFM 0406.

Genome sequencing was carried out using the Illumina HiSeq 2500 and Pacific Biosciences RS II sequencing platforms. Genomic DNA was isolated (ZR fungal/bacterial DNA mini prep kit, Zymo Research) and a paired-end library with bar coding was constructed (Nextera DNA sample preparation kit). The HiSeq paired-end data were processed and filtered, and a quality analysis was performed. This resulted in 6,564,821 reads with an average length of 509 bp (approx. 360× coverage). The quality of the Illumina FASTQ sequences was enhanced by trimming off low-quality bases, which was followed by *de novo* assembly using CLC Genomics Workbench version 7.0.4 to yield contigs. The optimal *k*-mer size was determined using KmerGenie (15). In addition, a 10-kb genomic sublibrary of strain IFM 0406 was produced and sequenced with PacBioSMRT technology. The resultant continuous long-read (CLR) data were filtered using the SMRT Analysis software suite. In summary, one run produced 113,652 filtered reads with average read lengths of 3,262 bp (approx. 40× coverage). The contigs were linked and placed into super-scaffolds based on the alignment of the PacBio CLR reads using BLASR (16) and SSPACE-LongRead scaffolder version 7.0.4 (17). The gapped regions within the super-scaffolds were closed using GapFiller version 1.10 (18).

The final gap-closed genome comprises three scaffolds with a total size of 9,276,856 bp and a G+C content of 68.5%. The genome contains 8,755 open reading frames, including 76 tRNA genes and nine rRNA operons. Bioinformatic analyses using antiSMASH version 3.0 (19) revealed that IFM 0406 possessed, besides the putative brasilicardin (14) and brasilinolide (20) gene cluster, 47 further orphan natural product biosynthesis gene clusters. Insight into the genome of strain IFM 0406 not only reveals a high potential to produce secondary metabolites, but will also enable and facilitate the heterologous expression and biotechnological production of brasilicardins in a nonpathogenic host system.

### Accession number(s).

This genome sequence has been deposited in EMBL/GenBank under the accession number LWGR00000000. The version described in this paper is the first version.
